# 

**Published:** 1878-10

**Authors:** 


*CONTENTS.*

| | Page | | Page |
|---|---|---|---|
| Temperance | 601 | Tea and Coffee | 622 |
| Vital Capacity | 609 | Milk | 623 |
| Eating and Drinking | 616 | Corns Cured | 624 |
| Scarlet Fever | 616 | Going South | 624 |
| Grow Beautiful | 620 | How to Live 100 Years | 626 |
| Aims of The Journal | 621 | | |

